# Knowledge, Attitude, and Perception Toward Evaluation of Acute Heart Failure Among Emergency Medicine Residents in Riyadh, Saudi Arabia

**DOI:** 10.7759/cureus.76500

**Published:** 2024-12-28

**Authors:** Saeed Nassar, Haneen Alkhanbashi, Ahmed M Alkhars, Tawfiq Almezeiny

**Affiliations:** 1 Department of Emergency Medicine, King Khalid University Hospital, Riyadh, SAU; 2 College of Medicine, Dar Al Uloom University, Riyadh, SAU

**Keywords:** acute heart failure, clinical assessment, confidence levels, diagnostic skills, medical residents

## Abstract

Background: Acute heart failure (AHF) poses significant challenges in clinical practice due to its varied clinical presentations and complex management strategies. Adequate confidence levels among medical residents in diagnosing and managing AHF are crucial for timely and effective patient care.

Study aim: This cross-sectional study aimed to assess medical residents' knowledge, attitude, and perception levels in diagnosing and managing AHF and explore factors influencing their confidence levels.

Methodology: A total of 52 medical residents from various specialties participated in the study. Based on different clinical parameters, participants completed a questionnaire assessing their confidence levels in diagnosing AHF. Descriptive statistics were used to summarize confidence levels, while chi-square tests explored associations between demographic/clinical factors and confidence levels.

Results: The majority of residents exhibited high confidence levels in diagnosing heart failure (HF) based on history and physical examination findings alone (22 (42.3%) and 16 (30.8%), respectively). However, confidence levels were lower when relying solely on laboratory tests and imaging modalities for diagnosis (17 (32.7%) and 18 (34.6%), respectively). Factors such as symptoms (e.g., dizziness) and availability of ultrasound equipment were associated with higher confidence levels (p < 0.05). However, demographic factors such as age, gender, and residency year did not significantly influence confidence levels (p > 0.05).

Conclusion: Our study highlights variations in confidence levels among medical residents in diagnosing and managing AHF. While clinical assessment remains paramount, targeted educational interventions focusing on interpreting diagnostic tests and imaging findings are warranted to enhance residents' confidence levels and improve patient care.

## Introduction

Acute decompensated heart failure (ADHF) represents a critical challenge in emergency medicine, often necessitating urgent evaluation and management to mitigate potentially life-threatening complications [1,2]. As frontline healthcare providers, emergency medicine residents play a pivotal role in the timely recognition and assessment of ADHF in the emergency department (ED) [3]. However, the accurate diagnosis of ADHF requires a comprehensive understanding of its pathophysiology, clinical presentation, and diagnostic modalities, including biomarkers and point-of-care ultrasound (POCUS) [4].

Heart failure (HF) is a multifaceted clinical syndrome characterized by the heart's inability to adequately pump blood to meet the body's metabolic demands. It encompasses a spectrum of symptoms ranging from dyspnea and fatigue to fluid retention and peripheral edema [5,6]. With the prevalence of HF on the rise globally, particularly in urban centers such as Riyadh, Saudi Arabia, emergency healthcare systems are faced with an increasing burden of ADHF cases, emphasizing the importance of proficient evaluation by emergency medicine residents [7,8].

Distinguishing ADHF from other causes of acute dyspnea can be challenging due to overlapping clinical features and comorbidities [9]. While traditional diagnostic methods such as history-taking, physical examination, and chest radiography remain fundamental, biomarkers such as B-type natriuretic peptide (BNP) and its N-terminal prohormone (NT-proBNP) have emerged as valuable adjuncts in aiding diagnosis and risk stratification [1-3].

Furthermore, point-of-care ultrasound (POCUS) has revolutionized the evaluation of patients with acute dyspnea, offering real-time visualization of cardiac and pulmonary anatomy and function at the bedside [10]. Lung ultrasound, in particular, has demonstrated high sensitivity and specificity in detecting pulmonary edema, a hallmark feature of ADHF, thereby facilitating prompt diagnosis and appropriate management decisions [11].

Despite the growing body of evidence supporting the utility of biomarkers and POCUS in the evaluation of ADHF [12-14], there remains a paucity of data on emergency medicine residents' knowledge, attitudes, and perceptions toward these diagnostic modalities in Riyadh, Saudi Arabia. Understanding their level of familiarity, confidence, and proficiency in utilizing these tools is paramount for enhancing diagnostic accuracy and optimizing patient outcomes in the ED setting [3].

Study aim

The aim of this study is to assess the knowledge, attitude, and perception of emergency medicine residents toward the evaluation of acute heart failure (AHF) in Riyadh, Saudi Arabia.

Study objectives

The objectives of this study are to evaluate the level of understanding of AHF diagnosis through clinical and investigative assessment among emergency medicine residents in Riyadh, Saudi Arabia; to assess the competency of emergency medicine residents in utilizing point-of-care ultrasound, particularly lung ultrasound, as an adjunctive tool in diagnosing AHF; to determine the attitudes of emergency medicine residents toward the importance and utility of biomarkers, such as NT-proBNP, in AHF evaluation; and to explore the perceptions of emergency medicine residents regarding the feasibility and practicality of integrating advanced diagnostic modalities, such as point-of-care ultrasound, into routine clinical practice for AHF assessment.

## Materials and methods

This cross-sectional observational study was conducted to assess the knowledge, attitude, and perception of emergency medicine residents toward the evaluation of acute heart failure (AHF) in Riyadh, Saudi Arabia. The study was carried out at King Saud University Medical City, a tertiary care academic medical center in Riyadh, chosen for its status as a major referral center for emergency medical care, ensuring a diverse and representative sample of emergency medicine residents. Data collection spanned six months, from January to June 2023. Participants included all residents enrolled in the Saudi Board of Emergency Medicine (SBEM) training program in Riyadh. The inclusion criteria encompassed residents at all levels of training within the SBEM program, and no specific exclusion criteria were applied, ensuring a comprehensive representation of eligible residents.

The primary data collection tool was a structured questionnaire developed by the research team. The questionnaire consisted of three sections: demographic information, knowledge assessment, and attitude and perception assessment. The demographic section collected basic information such as age, gender, level of training, and years of experience in emergency medicine. The knowledge assessment section included multiple-choice questions (MCQs) designed to evaluate participants' understanding of AHF pathophysiology, clinical presentation, and diagnostic approaches. The attitude and perception section utilized Likert scale questions to assess participants' views on the importance of biomarkers and point-of-care ultrasound (POCUS) in AHF evaluation, with participants rating their agreement with statements related to the utility and feasibility of these diagnostic modalities. The questionnaire underwent rigorous validation to ensure its validity and reliability. Content validity was established through expert review by emergency medicine physicians and researchers familiar with AHF evaluation. A pilot test was conducted among a sample of emergency medicine residents to assess the clarity, comprehensibility, and relevance of the items. Internal consistency reliability was evaluated using Cronbach's alpha coefficient for the knowledge assessment and Likert scale items, demonstrating high internal consistency (Cronbach's alpha > 0.70) across all sections, indicating satisfactory reliability. Data from the questionnaires were entered into a secure electronic database using IBM SPSS Statistics version 26 (IBM Corp., Armonk, NY). The data entry process involved double-entry verification to minimize errors and ensure accuracy. The dataset was cleaned and checked for outliers and missing values before analysis. Descriptive statistics were then generated to summarize demographic characteristics and responses to the questionnaire.

Ethical approval was obtained from the Institutional Review Board (IRB) of King Saud University Medical City (approval number: E-22-7069). Informed consent was secured from all participants, who were assured of the confidentiality and anonymity of their responses. Participation was voluntary, and participants retained the right to withdraw at any time without repercussion. Data confidentiality was maintained by assigning unique identifiers to participants, and only authorized research personnel had access to the collected data.

## Results

Table 1 summarizes the demographic characteristics of the 52 emergency medicine residents who participated in the study, showcasing a predominantly younger cohort. The age distribution reveals that most residents (n=28 (53.8%)) are within the 25-27 age group, while 24 (46.2%) fall in the 28-34 age range. Gender analysis shows a male predominance, with 36 (69.2%) male patients compared to 16 (30.8%) female patients. Residency year distribution demonstrates a balanced representation across training levels: 18 (34.6%) residents are in postgraduate year 1 (PGY1), nine (17.3%) in PGY2, 14 (26.9%) in PGY3, and 11 (21.2%) in PGY4. Furthermore, hospital affiliation highlights that the majority of participants (n=31 (59.6%)) are associated with university tertiary care hospitals, followed by 19 (36.5%) from governmental tertiary care hospitals, and two (3.8%) from private tertiary care hospitals. This distribution reflects diverse training environments and provides insight into the varying experiences of the participants. Transitioning from these findings, subsequent analyses explore the implications of these demographics on the study outcomes.

**Table 1:** Characters of the participating residents (N=52) PGY: postgraduate year

Parameter		Frequency (%)
Age, years	25-27	28 (53.8%)
	28-34	24 (46.2%)
Gender	Female	16 (30.8%)
	Male	36 (69.2%)
Residency year	PGY1	18 (34.6%)
	PGY2	9 (17.3%)
	PGY3	14 (26.9%)
	PGY4	11 (21.2%)
Type of hospital	Governmental tertiary care hospital	19 (36.5%)
	Private tertiary care hospital	2 (3.8%)
	University tertiary care hospital	31 (59.6%)

Table 2 highlights the participating residents' understanding of acute heart failure (AHF) diagnosis and management, illustrating varying levels of recognition for specific and non-specific markers. Among symptoms, orthopnea is most widely recognized, with 49 (94.2%) residents identifying it as indicative of HF, followed closely by paroxysmal nocturnal dyspnea (n=46 (88.5%)) and ankle swelling (n=43 (82.7%)). In contrast, abdominal bloating (n=8 (15.4%)) and anorexia (n=3 (5.8%)) are less frequently associated with HF. Regarding clinical signs, elevated jugular venous pressure (n=42 (80.4%)) and pulmonary crackles (n=47 (90.2%)) are commonly acknowledged, whereas confusion (n=2 (3.9%)) and the presence of a third heart sound (n=14 (27.5%)) are less recognized indicators.

**Table 2:** Understanding of AHF diagnosis and management by the participating residents (N=52) AHF: acute heart failure, HF: heart failure, ED: emergency department, E-FAST: extended focused assessment with ultrasonography in trauma

Parameter		Frequency (%)
Symptoms considered as specific or non‐specific markers for HF	Abdominal bloating	8 (15.4%)
	Ankle swelling	43 (82.7%)
	Anorexia	3 (5.8%)
	Breathlessness on exertion	44 (84.6%)
	Chest pain	19 (36.5%)
	Chronic cough	16 (30.8%)
	Dizziness	5 (9.6%)
	Fatigue	26 (50%)
	Nocturia	8 (15.4%)
	Orthopnea	49 (94.2%)
	Palpitations	10 (19.2%)
	Paroxysmal nocturnal dyspnea	46 (88.5%)
	Reduced exercise tolerance	36 (69.2%)
	Sputum	6 (11.5%)
Signs considered as specific or non‐specific markers for HF	Ankle edema	45 (88.2%)
	Confusion	2 (3.9%)
	Displaced apex beat	8 (15.7%)
	Elevated jugular venous pressure	41 (80.4%)
	Heart murmur	7 (13.7%)
	Pulmonary crackles	46 (90.2%)
	Tachycardia	18 (35.3%)
	Third heart sound	14 (27.5%)
Diagnostic tests done when suspecting HF	Assessment of medical history, comorbidities, and current medications	49 (94.2%)
	Assessment of signs and symptoms	49 (94.2%)
	Chest X‐ray	50 (96.2%)
	Coronary angiography	3 (5.8%)
	Echocardiogram	44 (84.6%)
	Electrocardiogram	39 (75%)
	Laboratory tests for renal function and electrolytes and to detect comorbidity	45 (86.5%)
	Liver function tests	28 (53.8%)
	Renal function and electrolytes	38 (73.1%)
	Serum natriuretic peptides	47 (90.4%)
	Thyroid function tests	3 (5.8%)
Is your emergency department equipped with an ultrasound machine for use by residents of emergency medicine?	No	1 (1.9%)
	Yes	51 (98.1%)
Which of the following companies were your ultrasound machines purchased from?	General Electric	1 (2%)
	Philips	11 (21.6%)
	Siemens	5 (9.8%)
	Sonosite	35 (68.6%)
Does your emergency department provide ultrasound training for emergency medicine residents?	Yes	52 (100%)
If yes, what kind of ultrasound education methods does your ED provide?	Hands-on experience, including bedside teaching	45 (86.5%)
	Send to another department or hospital	4 (7.7%)
	Workshops (courses)	3 (5.8%)
Is it mandatory for your program to do ultrasound training?	No	2 (3.8%)
	Yes	50 (96.2%)
Who funds this training?	Residency training program	48 (92.3%)
	Trainee	4 (7.7%)
For which of the following purposes were you trained to use point-of-care ultrasound?	Echocardiography	41 (85.4%)
	E-FAST	45 (93.8%)
	Lung ultrasound	35 (72.9%)
	Ultrasound-guided central line insertion	37 (77.1%)
	Ultrasound-guided peripheral line insertion	22 (45.8%)
How often do you use point-of-care ultrasound and echocardiography in clinical practice?	>Once per day	9 (17.3%)
	Daily	21 (40.4%)
	Once per week	3 (5.8%)
	3-4 times per week	14 (26.9%)
	Twice per week	5 (9.6%)
How often has point-of-care ultrasound and echocardiography changed your clinical decision-making per week?	>Once per day	2 (3.8%)
	Daily	7 (13.5%)
	Once per week	10 (19.2%)
	3-4 times per week	13 (25%)
	Twice per week	20 (38.5%)
Are you in agreement that point-of-care echocardiography can improve the quality of patient care?	Agree	14 (26.9%)
	Neutral	2 (3.8%)
	Strongly agree	33 (63.5%)
	Strongly disagree	3 (5.8%)

Diagnostic tests reveal a strong preference for chest X-ray (n=50 (96.2%)) and serum natriuretic peptides (n=47 (90.4%)), whereas coronary angiography (n=3 (5.8%)) and thyroid function tests (n=3 (5.8%)) are seldom utilized. Nearly all emergency departments (n=51 (98.1%)) have ultrasound machines available, predominantly the Sonosite brand (n=36 (68.6%)), with comprehensive ultrasound training provided to all residents (n=52 (100%)), primarily through hands-on experience (n=45 (86.5%)). Mandatory ultrasound training is funded by the residency program for most residents (n=50 (96.2%)), with echocardiography (n=45 (85.4%)) and extended focused assessment with ultrasonography in trauma (E-FAST) (n=49 (93.8%)) being the primary training applications.

Point-of-care ultrasound and echocardiography are regularly employed in clinical practice, demonstrating their critical role in patient care. Most residents (n=47 (90.4%)) agree or strongly agree that point-of-care echocardiography enhances patient care quality, underscoring its recognized importance in emergency medicine training and decision-making. These findings emphasize the integration of ultrasound technologies as a fundamental component in the diagnosis and management of AHF.

Table 3 explores the confidence levels of emergency medicine residents in diagnosing and managing acute heart failure (AHF) across various clinical parameters, assessed on a scale from 1 (low confidence) to 7 (high confidence). The findings reveal that residents exhibit the highest confidence when diagnosing HF based on history alone, with 22 (42.3%) individuals reporting high confidence. Similarly, confidence levels remain relatively strong when diagnosing HF through physical examination findings alone and when considering comorbidities, as evidenced by 16 (30.8%) and 19 (36.5%) residents, respectively, expressing high confidence.

**Table 3:** Items of assessing the confidence of residents in diagnosing and managing AHF (N=52) AHF: acute heart failure, HF: heart failure, CXR: chest X‐ray, US: ultrasound

Parameter	1 (low)	2	3	4	5	6	7 (high)
How confident are you in diagnosing HF based on history alone?	1 (1.9%)	2 (3.8%)	3 (5.8%)	7 (13.5%)	22 (42.3%)	10 (19.2%)	7 (13.5%)
How confident are you in diagnosing HF based on physical examination findings alone?	0 (0%)	1 (1.9%)	3 (5.8%)	9 (17.3%)	16 (30.8%)	12 (23.1%)	11 (21.2%)
How confident are you in diagnosing HF based on laboratory tests and CXR alone?	0 (0%)	1 (1.9%)	5 (9.6%)	17 (32.7%)	12 (23.1%)	14 (26.9%)	3 (5.8%)
How confident are you in diagnosing HF based on US (echo and lung US) alone?	2 (3.8%)	1 (1.9%)	2 (3.8%)	6 (11.5%)	18 (34.6%)	14 (26.9%)	9 (17.3%)
How confident are you in diagnosing HF in patient with comorbidities?	2 (3.8%)	1 (1.9%)	6 (11.5%)	14 (26.9%)	19 (36.5%)	7 (13.5%)	3 (5.8%)
How confident are you in diagnosing HF with reduced ejection fraction?	1 (1.9%)	1 (1.9%)	3 (5.8%)	6 (11.5%)	18 (34.6%)	14 (26.9%)	9 (17.3%)
How confident are you with identifying atrial fibrillation?	0 (0%)	0 (0%)	1 (1.9%)	6 (11.5%)	9 (17.3%)	13 (25%)	23 (44.2%)
How confident are you with identifying left bundle branch block?	1 (1.9%)	0 (0%)	4 (7.7%)	7 (13.5%)	5 (9.6%)	11 (21.2%)	24 (46.2%)
How confident are you with identifying right bundle branch block?	0 (0%)	2 (3.8%)	4 (7.7%)	8 (15.4%)	13 (25%)	4 (7.7%)	21 (40.4%)
How confident are you with identifying left ventricular hypertrophy?	1 (1.9%)	0 (0%)	7 (13.5%)	9 (17.3%)	12 (23.1%)	16 (30.8%)	7 (13.5%)

In contrast, lower confidence levels are observed when relying exclusively on laboratory tests, chest X-rays, or ultrasound (echo and lung US) for diagnosis, with fewer residents reporting high confidence in these approaches. Specific areas such as identifying atrial fibrillation or left bundle branch block also demonstrate variability in confidence, highlighting potential gaps in diagnostic proficiency.

These results underscore the need for focused educational initiatives to enhance residents' confidence and skills in utilizing diagnostic modalities, particularly those involving imaging and complex electrocardiographic findings. Strengthening training in these areas is essential to support comprehensive and accurate AHF diagnosis and management in emergency settings.

Table 4 presents an aggregate evaluation of the residents' confidence in diagnosing and managing acute heart failure (AHF), offering an overarching perspective on the cohort's confidence levels. The findings indicate that the majority of residents (n=35 (67.3%)) exhibit a high level of confidence, reflecting a robust self-assessment of their diagnostic and management capabilities. This is further substantiated by the mean confidence score of 52.4 ± 8.1, with scores ranging from 29 to 66, signifying an overall favorable confidence profile among the participants.

**Table 4:** Summary score of the residents' confidence in diagnosing and managing AHF (N=52) AHF: acute heart failure, SD: standard deviation

Parameter		Frequency (%)
Confidence score categories	Low	4 (7.7%)
	Average	13 (25.0%)
	High	35 (67.3%)
Confidence score	Mean ± SD (min-max)	52.4 ± 8.1 (29-66)

Despite this positive trend, a small subset of residents (n=4 (7.7%)) falls into the low confidence category, highlighting some degree of variability within the cohort. These findings suggest that while most residents feel well-prepared, targeted efforts may be necessary to address the specific needs of those with lower confidence levels, ensuring a uniformly high standard of diagnostic and management proficiency across the group.

Table 5 examines the relationship between confidence score categories and various demographic and clinical factors among residents, using chi-square tests to assess statistical significance. The findings indicate no significant associations between confidence score categories and demographic variables such as age (χ² = 4.669, p = 0.097), gender (χ² = 4.341, p = 0.114), residency year (χ² = 9.619, p = 0.142), or type of hospital (χ² = 2.811, p = 0.590).

**Table 5:** Summary confidence score categories in association with characters and AHF understanding among residents (N=52) AHF: acute heart failure, HF: heart failure, ED: emergency department, PGY: postgraduate year, E-FAST: extended focused assessment with ultrasonography in trauma

Parameter		Confidence score categories			Total	X^2^	P-value
		Low	Average	High			
Age, years	25-27	4 (14.3%)	8 (28.6%)	16 (57.1%)	28 (100%)	4.669	0.097
	28-34	0 (0%)	5 (20.8%)	19 (79.2%)	24 (100%)		
Gender	Female	1 (6.3%)	7 (43.8%)	8 (50%)	16 (100%)	4.341	0.114
	Male	3 (8.3%)	6 (16.7%)	27 (75%)	36 (100%)		
Residency year	PGY1	4 (22.2%)	3 (16.7%)	11 (61.1%)	18 (100%)	9.619	0.142
	PGY2	0 (0%)	3 (33.3%)	6 (66.7%)	9 (100%)		
	PGY3	0 (0%)	5 (35.7%)	9 (64.3%)	14 (100%)		
	PGY4	0 (0%)	2 (18.2%)	9 (81.8%)	11 (100%)		
Type of hospital	Governmental tertiary care hospital	2 (10.5%)	3 (15.8%)	14 (73.7%)	19 (100%)	2.811	0.590
	Private tertiary care hospital	0 (0%)	0 (0%)	2 (100%)	2 (100%)		
	University tertiary care hospital	2 (6.5%)	10 (32.3%)	19 (61.3%)	31 (100%)		
Symptoms considered as specific or non‐specific markers for HF	Ankle swelling	4 (9.3%)	10 (23.3%)	29 (67.4%)	43 (100%)	1.140	0.565
	Anorexia	0 (0%)	2 (66.7%)	1 (33.3%)	3 (100%)	3.002	0.223
	Breathlessness on exertion	3 (6.8%)	11 (25%)	30 (68.2%)	44 (100%)	0.317	0.854
	Chest pain	2 (10.5%)	5 (26.3%)	12 (63.2%)	19 (100%)	0.410	0.815
	Chronic cough	3 (18.8%)	2 (12.5%)	11 (68.8%)	16 (100%)	5.125	0.077
	Dizziness	2 (40%)	1 (20%)	2 (40%)	5 (100%)	8.175	0.017
	Fatigue	3 (11.5%)	6 (23.1%)	17 (65.4%)	26 (100%)	1.105	0.575
	Nocturia	0 (0%)	4 (50%)	4 (50%)	8 (100%)	3.512	0.173
	Orthopnea	4 (8.2%)	12 (24.5%)	33 (67.3%)	49 (100%)	0.334	0.846
	Palpitations	1 (10%)	2 (20%)	7 (70%)	10 (100%)	0.223	0.895
	Paroxysmal nocturnal dyspnea	3 (6.5%)	12 (26.1%)	31 (67.4%)	46 (100%)	0.899	0.638
	Reduced exercise tolerance	4 (11.1%)	9 (25%)	23 (63.9%)	36 (100%)	1.981	0.371
	Sputum	1 (16.7%)	0 (0%)	5 (83.3%)	6 (100%)	2.665	0.264
	Abdominal bloating	0 (0%)	2 (25%)	6 (75%)	8 (100%)	0.810	0.667
Signs considered as specific or non‐specific markers for HF	Tachycardia	1 (5.6%)	5 (27.8%)	12 (66.7%)	18 (100%)	0.250	0.882
	Third heart sound	0 (0%)	5 (35.7%)	9 (64.3%)	14 (100%)	2.379	0.304
	Pulmonary crackles	3 (6.5%)	11 (23.9%)	32 (69.6%)	46 (100%)	1.200	0.549
	Heart murmur	1 (14.3%)	1 (14.3%)	5 (71.4%)	7 (100%)	0.849	0.654
	Elevated jugular venous pressure	2 (4.9%)	11 (26.8%)	28 (68.3%)	41 (100%)	2.283	0.319
	Confusion	0 (0%)	2 (100%)	0 (0%)	2 (100%)	6.240	0.044
	Displaced apex beat	1 (12.5%)	1 (12.5%)	6 (75%)	8 (100%)	0.958	0.619
	Ankle edema	3 (6.7%)	10 (22.2%)	32 (71.1%)	45 (100%)	2.207	0.332
Diagnostic tests done when suspecting HF	Assessment of medical history, comorbidities, and current medications	4 (8.2%)	12 (24.5%)	33 (67.3%)	49 (100%)	0.334	0.846
	Assessment of signs and symptoms	4 (8.2%)	11 (22.4%)	34 (69.4%)	49 (100%)	3.002	0.223
	Chest X‐ray	4 (8%)	12 (24%)	34 (68%)	50 (100%)	0.773	0.680
	Coronary angiography	0 (0%)	1 (33.3%)	2 (66.7%)	3 (100%)	0.334	0.846
	Echocardiogram	4 (9.1%)	11 (25%)	29 (65.9%)	44 (100%)	0.810	0.667
	Electrocardiogram	3 (7.7%)	8 (20.5%)	28 (71.8%)	39 (100%)	1.723	0.423
	Laboratory tests for renal function and electrolytes and to detect comorbidity	2 (4.4%)	11 (24.4%)	32 (71.1%)	45 (100%)	5.344	0.069
	Liver function tests	4 (14.3%)	6 (21.4%)	18 (64.3%)	28 (100%)	3.820	0.148
	Renal function and electrolytes	3 (7.9%)	7 (18.4%)	28 (73.7%)	38 (100%)	3.304	0.192
	Serum natriuretic peptides	4 (8.5%)	11 (23.4%)	32 (68.1%)	47 (100%)	0.967	0.617
	Thyroid function tests	1 (33.3%)	1 (33.3%)	1 (33.3%)	3 (100%)	3.355	0.187
Is your emergency department equipped with an ultrasound machine for use by residents of emergency medicine?	No	0 (0%)	1 (100%)	0 (0%)	1 (100%)	3.059	0.217
	Yes	4 (7.8%)	12 (23.5%)	35 (68.6%)	51 (100%)		
Which of the following companies were your ultrasound machines purchased from?	General Electric	0 (0%)	0 (0%)	1 (100%)	1 (100%)	0.495	0.781
	Philips	0 (0%)	4 (36.4%)	7 (63.6%)	11 (100%)	1.822	0.402
	Sonosite	4 (11.4%)	8 (22.9%)	23 (65.7%)	35 (100%)	2.180	0.336
	Siemens	0 (0%)	2 (40%)	3 (60%)	5 (100%)	0.967	0.617
Does your emergency department provide ultrasound training for emergency medicine residents?	Yes	4 (7.7%)	13 (25%)	35 (67.3%)	52 (100%)	NA	NA
If yes, what kind of ultrasound education methods does your ED provide?	Hands-on experience, including bedside teaching	4 (8.9%)	11 (24.4%)	30 (66.7%)	45 (100%)	0.749	0.945
	Send to another department or hospital	0 (0%)	1 (25%)	3 (75%)	4 (100%)		
	Workshops (courses)	0 (0%)	1 (33.3%)	2 (66.7%)	3 (100%)		
Is it mandatory for your program to do ultrasound training?	No	0 (0%)	0 (0%)	2 (100%)	2 (100%)	1.010	0.603
	Yes	4 (8%)	13 (26%)	33 (66%)	50 (100%)		
Who funds this training?	Residency training program	3 (6.3%)	12 (25%)	33 (68.8%)	48 (100%)	1.880	0.391
	Trainee	1 (25%)	1 (25%)	2 (50%)	4 (100%)		
For which of the following purposes were you trained to use point-of-care ultrasound?	E-FAST	3 (6.7%)	12 (26.7%)	30 (66.7%)	45 (100%)	0.849	0.654
	Lung ultrasound	3 (8.6%)	10 (28.6%)	22 (62.9%)	35 (100%)	0.969	0.616
	Echocardiography	4 (9.8%)	12 (29.3%)	25 (61%)	41 (100%)	3.640	0.162
	Ultrasound-guided central line insertion	3 (8.1%)	11 (29.7%)	23 (62.2%)	37 (100%)	1.681	0.431
	Ultrasound-guided peripheral line insertion	3 (13.6%)	8 (36.4%)	11 (50%)	22 (100%)	5.418	0.067
How often do you use point-of-care ultrasound and echocardiography in clinical practice?	>Once per day	0 (0%)	3 (33.3%)	6 (66.7%)	9 (100%)	11.807	0.160
	Daily	2 (9.5%)	4 (19%)	15 (71.4%)	21 (100%)		
	Once per week	0 (0%)	2 (66.7%)	1 (33.3%)	3 (100%)		
	3-4 times per week	1 (7.1%)	1 (7.1%)	12 (85.7%)	14 (100%)		
	Twice per week	1 (20%)	3 (60%)	1 (20%)	5 (100%)		
How often has point-of-care ultrasound and echocardiography changed your clinical decision-making per week?	>Once per day	0 (0%)	1 (50%)	1 (50%)	2 (100%)	2.525	0.961
	Daily	1 (14.3%)	2 (28.6%)	4 (57.1%)	7 (100%)		
	Once per week	1 (10%)	2 (20%)	7 (70%)	10 (100%)		
	3-4 times per week	1 (7.7%)	2 (15.4%)	10 (76.9%)	13 (100%)		
	Twice per week	1 (5%)	6 (30%)	13 (65%)	20 (100%)		
Are you in agreement that point-of-care echocardiography can improve the quality of patient care?	Agree	2 (14.3%)	5 (35.7%)	7 (50%)	14 (100%)	4.705	0.582
	Neutral	0 (0%)	1 (50%)	1 (50%)	2 (100%)		
	Strongly agree	2 (6.1%)	7 (21.2%)	24 (72.7%)	33 (100%)		
	Strongly disagree	0 (0%)	0 (0%)	3 (100%)	3 (100%)		

Conversely, clinical factors show a more substantial influence on confidence levels. Symptoms such as dizziness (χ² = 8.175, p = 0.017) and confusion (χ² = 6.240, p = 0.044) are significantly associated with higher confidence scores, possibly reflecting their perceived diagnostic value in clinical settings. Furthermore, access to ultrasound equipment and training emerges as a key factor, with residents from departments equipped with ultrasound machines reporting greater confidence levels. These findings emphasize the interplay between clinical experience, resource availability, and confidence, underscoring the importance of enhanced training and resource allocation to improve residents' diagnostic and management skills in AHF.

## Discussion

Acute heart failure (AHF) represents a critical condition characterized by the rapid onset or exacerbation of heart failure symptoms, often necessitating urgent medical attention. The syndrome encompasses a spectrum of clinical presentations, including dyspnea, fatigue, and fluid retention, and is classified based on ejection fraction into heart failure with reduced ejection fraction (HFrEF) and heart failure with preserved ejection fraction (HFpEF) [15,16]. With a global prevalence estimated at 1%-2% of adults in developed countries, AHF imposes a substantial burden on healthcare systems and contributes significantly to morbidity and mortality rates [15].

Our study revealed several important findings regarding the confidence levels of residents in diagnosing and managing AHF. First, the majority of residents exhibited high confidence levels in diagnosing HF based on history and physical examination findings alone. This finding is consistent with previous literature suggesting that clinical assessment plays a central role in the initial evaluation of HF patients [2,3,17]. However, confidence levels were lower when relying solely on laboratory tests and imaging modalities for diagnosis, highlighting the complexity and challenges associated with interpreting diagnostic tests in the context of AHF [3,16].

Second, our analysis showed variations in confidence levels among residents based on demographic and clinical factors. While age, gender, residency year, and hospital type did not significantly influence confidence levels, certain symptoms and signs, such as dizziness and confusion, were associated with higher confidence levels. These findings suggest that clinical presentations with clearer manifestations may enhance residents' confidence in diagnosing AHF. Additionally, the availability of ultrasound equipment and training appeared to positively influence confidence levels, underscoring the importance of hands-on experience and access to technology in medical education [1,3,9].

The findings of our study have several implications for clinical practice and medical education. Firstly, the high confidence levels observed in diagnosing HF based on history and physical examination emphasize the importance of strengthening clinical skills among residents. While advancements in technology have expanded diagnostic capabilities, a thorough clinical assessment remains the cornerstone of AHF diagnosis [2,8]. Therefore, medical education programs should prioritize training residents in history-taking and physical examination techniques to enhance diagnostic accuracy and confidence levels.

Secondly, the lower confidence levels observed in interpreting laboratory tests and imaging modalities underscore the need for targeted educational interventions in this area. Studies have shown that residents often lack proficiency in interpreting diagnostic tests, leading to diagnostic errors and delays in patient management [3]. Incorporating structured teaching sessions and case-based learning approaches focusing on the interpretation of laboratory and imaging findings may help bridge this gap and improve residents' diagnostic confidence in AHF.

The literature underscores the challenges associated with diagnosing AHF, particularly in the acute setting. Undifferentiated dyspnea poses a diagnostic dilemma, with studies reporting a level of uncertainty in nearly half of patients presenting with AHF symptoms [17]. The incorporation of essential laboratory investigations, such as N-terminal pro-B-type natriuretic peptide (NT-proBNP), has emerged as a valuable tool in AHF diagnosis, offering high sensitivity and specificity, particularly in younger patients with elevated BNP levels [18]. However, the interpretation of BNP levels must consider various clinical factors, including age, comorbidities, and hemodynamic status, to avoid misdiagnosis [18].

Moreover, the integration of point-of-care ultrasound (POCUS) has revolutionized the diagnostic approach to AHF, enabling rapid and accurate assessments at the bedside. Lung ultrasound, in particular, has emerged as a highly beneficial adjunct in diagnosing acute cardiogenic pulmonary edema, offering superior sensitivity and specificity compared to traditional clinical assessment, chest radiography, and BNP testing [19,20]. Studies have demonstrated the utility of lung ultrasound in identifying characteristic findings, such as comet tail B-lines, indicative of alveolar interstitial syndrome, facilitating prompt diagnosis and timely interventions [19,21]. Similarly, bedside echocardiography has shown promise in aiding AHF diagnosis, with studies highlighting its accuracy in detecting left ventricular systolic and diastolic dysfunction [22].

Our study contributes to the growing body of evidence highlighting the importance of comprehensive diagnostic approaches in AHF management. The observed variations in confidence levels among medical residents underscore the need for targeted educational interventions aimed at enhancing diagnostic skills and interpretation of diagnostic tests. Incorporating POCUS training into medical curricula may empower residents to confidently assess and manage AHF at the bedside, ultimately improving patient outcomes and reducing healthcare costs [23].

Future research should adopt longitudinal designs and larger sample sizes to validate our findings and explore additional factors influencing residents' confidence levels in diagnosing and managing AHF. Additionally, evaluating the long-term impact of educational interventions on residents' diagnostic skills and patient outcomes is warranted.

This study has certain limitations that should be considered when interpreting the findings. The cross-sectional design captures residents' confidence levels at a single point in time, which may not fully reflect changes as they progress through their training or gain additional clinical exposure. The sample size of 52 participants, while sufficient for exploratory analysis, may limit the broader applicability of the results to other resident populations or training settings. Considering the objectives of our study, we used a self-reported confidence scale as no objective assessment was done for the residents during their management of cases. The performance of based validation would give much insight and can be the subject of future research. Lastly, variations in training exposure and resources across specialties were not specifically accounted for, which could introduce subtle differences in confidence levels. Despite these considerations, the study provides important insights into areas for targeted educational interventions to enhance residents' preparedness in managing acute heart failure.

## Conclusions

In conclusion, our study highlights the importance of clinical skills training and targeted educational interventions in enhancing residents' confidence levels in diagnosing and managing AHF. While residents exhibit high confidence levels in clinical assessment, further efforts are needed to improve proficiency in interpreting diagnostic tests and imaging findings. By addressing these educational gaps and leveraging technological advancements, medical education programs can better prepare residents for the challenges of diagnosing and managing AHF in clinical practice. Additionally, the study reveals that the availability of diagnostic tools such as ultrasound plays a significant role in boosting residents' confidence. These findings suggest that improving access to such resources and incorporating them into routine training could further support residents' development of diagnostic skills. By refining educational strategies to address these areas, medical programs can enhance residents' readiness to manage AHF more effectively, ultimately improving patient outcomes.

## Appendices

Table 6 presents the structured questionnaire developed by the research team.

**Table 6:** Structured questionnaire developed by the research team PGY: postgraduate year, HF: heart failure, CXR: chest X‐ray, US: ultrasound, ED: emergency department, E-FAST: extended focused assessment with ultrasonography in trauma

Questionnaire	
Age:	
What is your gender?	Male/female
Which year of residency are you currently in?	PGY1/PGY2/PGY3/PGY4/others (specify):
In which type of hospital do you work?	University tertiary care hospital/governmental tertiary care hospital/private tertiary care hospital/non-university maximum care hospital/community hospital (bed capacity < 500)/other
In your clinical practice, which of the following indicators do you routinely assess or consider as specific or non‐specific markers for HF? Select all that apply.	Abdominal bloating/ankle swelling/anorexia/breathlessness on exertion/chest pain/chronic cough/dizziness/fatigue/forgetfulness/confusion/nocturia/orthopnea/palpitations/paroxysmal nocturnal dyspnea/reduced exercise tolerance/sputum
In your clinical practice, which of the following indicators do you routinely assess or consider as specific or non‐specific markers for HF? Select all that apply.	Tachycardia/third heart sound/pulmonary crackles/heart murmur/elevated jugular venous pressure/confusion/displaced apex beat/ankle edema
When assessing patients for suspected HF, which of the following do you carry out for your patients? Select all that apply.	Assessment of medical history, comorbidities, and current medications/assessment of signs and symptoms/chest X‐ray/coronary angiography/CT angiography/echocardiogram/electrocardiogram/laboratory tests for renal function and electrolytes and to detect comorbidity/liver function tests/renal function and electrolytes/serum natriuretic peptides/thyroid function tests
How confident are you in diagnosing HF based on history alone?	1-7
How confident are you in diagnosing HF based on physical examination findings alone?	1-7
How confident are you in diagnosing HF based on labs and CXR alone?	1-7
How confident are you in diagnosing HF based on US (echo and lung US) alone?	1-7
How confident are you in diagnosing HF in patient with comorbidities?	1-7
How confident are you in diagnosing HF with reduced ejection fraction?	1-7
How confident are you in diagnosing HF with reduced ejection fraction?	1-7
How confident are you with identifying atrial fibrillation?	1-7
How confident are you with identifying left bundle branch block?	1-7
How confident are you with identifying right bundle branch block?	1-7
How confident are you with identifying left ventricular hypertrophy?	1-7
Is your emergency department equipped with an ultrasound machine for use by residents of emergency medicine?	Yes/no
Which of the following companies were your ultrasound machines purchased from?	General Electric/Philips/Sonosite/Medison/Siemens/other (specify):
Does your emergency department provide ultrasound training for emergency medicine residents?	Yes/no
If yes, is it mandatory from your program to do ultrasound training?	Yes/no
If yes, what kind of ultrasound education methods does your ED provide?	Send to another department or hospital/hands-on experience, including bedside teaching/conferences or lectures/workshops (courses)
Who funds this training?	Trainee/residency training program/other (specify):
For which of the following purposes were you trained to use point-of-care ultrasound? (Check all those that apply.)	E-FAST/lung/ultrasound/echocardiograph/ultrasound-guided central line insertion/ultrasound-guided peripheral line insertion/other (specify):
How often do you use point-of-care ultrasound and echocardiography in clinical practice?	Never/once per week/twice per week/3-4 times per week/daily/>once per day
How often has point-of-care ultrasound and echocardiography changed your clinical decision-making per week?	Never/once per week/twice per week/3-4 times per week/daily/>once per day
Are you in agreement that point-of-care echocardiography can improve the quality of patient care?	Strongly agree/agree/neutral/disagree/strongly disagree
